# Expression of Dopamine Receptors in the Lateral Hypothalamic Nucleus and Their Potential Regulation of Gastric Motility in Rats With Lesions of Bilateral Substantia Nigra

**DOI:** 10.3389/fnins.2019.00195

**Published:** 2019-03-14

**Authors:** Yan-Li Yang, Xue-Rui Ran, Yong Li, Li Zhou, Li-Fei Zheng, Yu Han, Qing-Qing Cai, Zhi-Yong Wang, Jin-Xia Zhu

**Affiliations:** ^1^Xinxiang Key Laboratory of Molecular Neurology, Department of Human Anatomy, School of Basic Medical Sciences, Xinxiang Medical University, Xinxiang, China; ^2^Department of Physiology and Pathophysiology, School of Basic Medical Sciences, Capital Medical University, Beijing, China; ^3^Department of Gastroenterology, The First Affiliated Hospital of Xinxiang Medical University, Xinxiang, China

**Keywords:** dopamine receptor, lateral hypothalamic nucleus, orexin receptor 1, gastroparesis, Parkinson’s disease

## Abstract

Most Parkinson’s Disease (PD) patients experience gastrointestinal (GI) dysfunction especially the gastroparesis, but its underlying mechanism is not clear. We have previously demonstrated that the neurons in the substantia nigra (SN) project to the lateral hypothalamic nucleus (LH) and the dorsal motor nucleus of vagus (DMV) receives the neural projection from LH by the means of anterograde and retrograde neural tracing technology. Orexin A (OXA) is predominately expressed in the LH. It has been reported that OXA can alter the gastric motility through the orexin receptor 1 (OX1R) in DMV. We speculated that this SN-LH-DMV pathway could modulate the motility of stomach because of the important role of LH and DMV in the regulation of gastric motility. However, the distribution and expression of dopamine receptors (DR) in the LH is unknown. In the present study, using a double-labeling immunofluorescence technique combined with confocal microscopy, we significantly extend our understanding of the SN-LH-DMV pathway by showing that (1) a considerable quantity of dopamine receptor 1 and 2 (D1 and D2) was expressed in the LH as well as the OX1R was expressed in the DMV; (2) Nearly all of the D1-immuoreactve (IR) neurons were also OXA-positive while only a few neurons express both D2 and OXA in the LH, and the DR-positive neurons were surrounded by the dopaminergic neural fibers; In the DMV, OX1R were colocalized with choline acetyltransferase (ChAT)-labeled motor neurons; (3) When the gastroparesis was induced by the destruction of dopaminergic neurons in the SN, the decreased expression of D1 and OXA was observed in the LH as well as the reduced OX1R and ChAT expression in the DMV. These findings suggest that SN might regulate the function of OXA-positive neurons via D1 receptor, which then affect the motor neurons in the DMV through OX1R. If the SN is damaged the vagal pathway would be affected, which may lead to gastric dysfunction. The present study raises the possibility that the SN-LH-DMV pathway can regulate the movement of stomach.

## Introduction

Parkinson’s disease (PD) is characterized by loss of dopaminergic neurons in the substantia nigra (SN) and decrease of dopamine level in the striatum of basal ganglia (Lees et al., 2009). Over 80% of PD patients experience gastrointestinal (GI) dysfunctions including gastroparesis (Jost and Eckardt, 2003; Jost, 2010). Accumulating evidence demonstrates that cholinergic neuron degeneration contributes to gastroparesis in PD (Travagli and Anselmi, 2016). We previously have reported the reduced cholinergic markers in the dorsal motor nucleus of vagus (DMV) and gastric muscularis in rats with bilateral SN lesion by 6-hydroxydopamine (6-OHDA). However, it is unknown how the SN influences the DMV.

The lateral hypothalamic nucleus (LH) in diencephalon has been identified as an important brain region that innervates multiple brain regions and regulates many important physiological processes including feeding, reward behaviors and autonomic function (Berthoud and Munzberg, 2011). Orexin (OX) neurons are primarily located in the LH (Sakurai et al., 1998; Takahashi et al., 2015). The OX neuropeptide family consists of orexin A (OXA) and B (OXB), which are encoded by the same pre-mRNA (Date et al., 1999; Sakurai et al., 1999). OXA regulates food intake (Dube et al., 1999) and gastric emptying in rats (Ehrstrom et al., 2005; Bulbul et al., 2010b). The OXA neurons stimulation or destruction will alter the movement of the stomach (Guo et al., 2018). Both the dopamine receptor 1 and 2 (D1 and D2) mRNA were reported to be expressed in LH neurons. OX neurons regulate GI functions through the brain-gut axis (Kirchgessner, 2002; Kukkonen et al., 2002). Microinjection of OXA into the DMV increased intragastric pressure and antral motility in anesthetized rats (Krowicki et al., 2002). Thus, OXA may regulate GI motility through the DMV (Grabauskas and Moises, 2003).

The DMV regulates upper GI functions, such as gastric motility. The orexin receptor 1 (OX1R) is highly expressed in the neurons of DMV, especially in the preganglionic neurons that innervate the stomach (Krowicki et al., 2002; Bulbul et al., 2010a). We previously have demonstrated that the SN and the DMV can contact with each other indirectly through the LH. By means of anterograde and retrograde neural tracing technology, we found that the neurons in the SN can project to the LH and the DMV receives neural projection from the LH (Wang et al., 2014). It is reported that a large number of OX neurons were lost in the LH of PD patients (Thannickal et al., 2007, 2008).

Based on the above results, we speculated that the OXA neurons in the LH could be regulated by dopaminergic projections from the SN through D1 or D2, destruction of the SN might change the expression of dopamine receptors (DR) in the LH and then orexin receptor (OXR) in the DMV. In the present study, double-labeling immunofluorescence procedures were performed to detect the distribution of tyrosine hydroxylase (TH), D1 and D2 in the LH and their co-localization with OXA neurons. The alternations of D1, D2, and OXA in the LH, and OX1R and choline acetyltransferase (ChAT) in the DMV were observed in the rats with bilateral SN injection of 6-OHDA. The present study may provide morphological evidences for DA/DR and OXA/OXR promoting gastric motility through SN-LH-DMV pathway.

## Materials and Methods

### Animals

Twenty-five adult male Sprague-Dawley rats (180–220 g) were purchased from Beijing Vital River Laboratory Animal Technology Co., Ltd. All experiments were performed in accordance with the guidelines established by the National Institutes of Health (NIH, United States) and were approved by the Animal Care and Use Committee of Xinxiang Medical University, Xinxiang, China. All efforts were made to minimize animal suffering, and the minimal number of animals necessary to produce reliable scientific data was used.

### 6-OHDA Animal Models

The methods used have been previously described (Zheng et al., 2014). Rats were anesthetized by intraperitoneal injection with chloral hydrate (0.4 g/kg) and placed on stereotaxic instrument. Two small areas of the skull were exposed (coordinates: AP, -5.6 mm; ML, ± 2.0 mm; DV, -7.5 mm), and 6-OHDA (4 μg in 2 μl of 0.9% saline containing 0.05% ascorbic acid) was injected with a 10 μl Hamilton syringe. Control groups were injected with 0.05% ascorbic acid/saline. The rats injected with 6-OHDA in the SN were referred to as 6-OHDA rats. Subsequent experiments were conducted at 6 weeks after 6-OHDA administration.

### Tissue Preparation

After deep anesthetization with chloral hydrate, rats received a thoracotomy and were perfused through the left ventricle with 250 ml of saline followed by 250 ml 4% paraformaldehyde in 0.01 M PBS (pH 7.4). The brains were immediately removed and immersed into 4% paraformaldehyde for a 12 h post-fixation period and then placed in 30% sucrose in 0.01 M PBS (pH 7.4) for at least 48 h until the dehydration achieved. The serial coronal sections which were 20 μm in thickness were made with a cryostat (Leica CM1850, St. Gallen, Switzerland). The tissue sections were air-dried overnight at room temperature and then stored at -80°C. In some experiments, the samples of dorsal medulla were collected (as descripted in our previous reports) on ice and immediately frozen in liquid nitrogen.

### Immunofluorescence Staining

The methods used have been described previously (Wang et al., 2016). The brain sections were permeabilized with PBS containing 0.3% Triton X-100, then blocked for unspecific binding with 5% goat serum for 30 min. Sections were then incubated overnight in a mixture of two primary antibodies derived from different host species for 12–16 h at 4°C (Table 1, TH/D1, TH/D2, OXA/TH, OXA/D1, OXA/D2, and OX1R/ChAT) and then incubated with the secondary antibodies for 1 h at room temperature. Micrographs were obtained using a confocal microscope (Olympus, FV1000).

**Table 1:** Antibodies used in the immunofluorescent study.

Antigen	Antibody	Dilution	Source/Catalog No.
TH	Mouse monoclonal	1:5000	Sigma/T1299
TH	Rabbit polyclonal	1:500	Abcam/ab112
OXA	Mouse monoclonal	1:50	Santa cruz/SC-80263
ChAT	Mouse monoclonal	1:100	Abcam/ab35948
OX1R	Rabbit polyclonal	1:250	Abcam/ab68718
D1	Rabbit polyclonal	1:100	Alomone/ADR-001
D2	Rabbit polyclonal	1:100	Alomone/ADR-002
Alexa fluor 488-labeled anti-mouse IgG	Goat	1:500	Beyotime/A0428
Alexa fluor 488-labeled anti-rabbit IgG	Goat	1:500	Beyotime/A0423
Cy3-labeled anti-mouse IgG	Goat	1:500	Beyotime/A0521
Cy3-labeled anti-rabbit IgG	Goat	1:500	Beyotime/A0516

*TH, tyrosine hydroxylase; ChAT, choline acetyltransferase; D1, dopamine 1 receptor; D2, dopamine 2 receptor; OXA, orexin A; OX1R, orexin receptor 1*.

The OXA-IR, D1R-IR, and D2R-IR neurons in the LH were counted from every three LH-containing section per animal (*n* = 3). Five different fields (150 pixel ^∗^150 pixel) of each section (512 pixel ^∗^512 pixel, 200×) were selected randomly for the neuron count. The average number of neurons in each section was calculated from nine sections of three rats in total. To reduce the counting error, the number of line pressing cells was only counted on one side and moves arduously.

### Western Blot Analysis

As in our previous reports (Zheng et al., 2014), tissues were homogenized in 300 μl cold lysis buffer supplemented with protease inhibitors for protein extraction. Proteins (100 μg) were loaded in a 10% SDS-PAGE gel and transferred onto a nitrocellulose membrane (NC membrane, Millipore, United States) at 4°C. After blocking with 10% non-fat dry milk in TBST for 2 h, the membranes were incubated with primary antibodies against ChAT (Rabbit polyclonal, Proteintech/20747-1-AP, 1:1,000), OX1R (Rabbit polyclonal, Abcam/ab68718, 1:500), or GAPDH (Rabbit polyclonal, Sigma/G9545, 1:5,000) overnight at 4°C. After washing, the membranes were incubated with horseradish-peroxidase-conjugated IgG (Pierce, Rockford, IL, United States) for 1 h at room temperature, then washed in TBST. Finally, the membranes were scanned with an Odyssey Infrared Imager (LI-COR, NE, United States), and analyzed using Odyssey software (version 1.2).

### Gastric Emptying

The methods used have been described previously (Zheng et al., 2014). Following a 24-h fast, a 2.5 ml barium meal was administrated to each rat through oral gavage. Plain radiographs of the GI tract were obtained using a KODAK *in vivo* Imaging System FX with the focus distance manually fixed to 50 ± 1 cm. The exposure time was adjusted to 30 s. Images were recorded at different time points (30, 60, and 90 min) after the consumption of the barium meal. Gastric emptying (GE) was calculated according to the following formula:

GE(%)=[1−(barium meal at Time 90 min/barium meal at Time zero)]×100.

### Anterograde Tracing

Approximately 4 μl of 20% Biotinylated Dextran Amines (BDA) (Eugene/n7167) (3 mg dissolved in 15 μl 0.01 M PBS, pH 7.4) was injected into the left SN (coordinates: AP, -5.6 mm; ML, -2.0 mm; DV, -7.5 mm) with the same Hamilton microsyringe. Seven days later, the animals were killed by decapitation, and the brains were removed for detection of the anterograde tracing fibers.

The sections from the anterograde tracing group were processed for observation of the injection site in the SN and the distribution of BDA-labeled fibers in the diencephalon and brainstem. The sections were incubated in 0.3% Triton X-100 in 0.01 M PBS (pH 7.6) for 15 min prior to incubation in fluorescent isothiocyanate (TEX RED)-labeled avidin D (1:200,A-2001,Vector Laboratories, Burlingame, CA, United States) at room temperature for 2 h. After incubation, all sections were rinsed in 0.01 M PBS, mounted onto gelatin-coated glass slides, air-dried, and cover-slipped with a mixture of 50% (v/v) glycerin and 2.5% (w/v) triethylene diamine (anti-fadingagent) in 0.01 M PBS. The injection sites and distribution of BDA-labeled fibers were examined under a fluorescence microscope.

### Statistical Analysis

The values are presented as the means ± S.E.M. (standard error of the mean) from at least three independent experiments; “n” refers to the number of animals or tissue samples from different animals. Statistical analysis was conducted using unpaired *t*-tests. The level of significance was set at *P* < 0.05.

## Results

### Neural Projection From the SN to the LH and the Distribution of TH-, D1-, D2-, and OXA-IR Neurons and Their Colocalization in the LH

**Figure 1:**
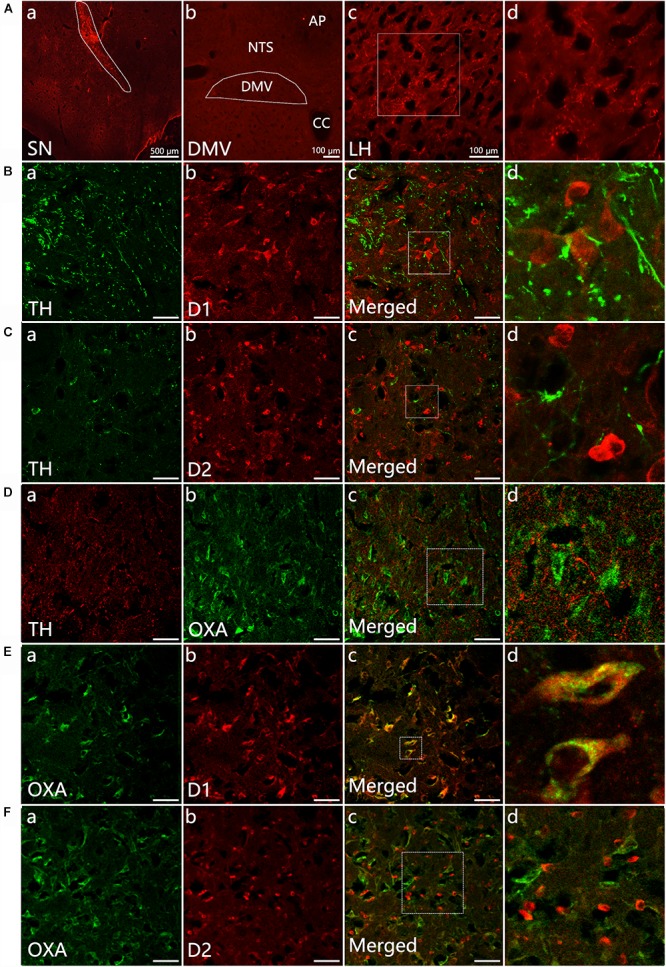
Neural projection from the SN to the LH and distribution of TH-, D1-, D2- and OXA-IR neurons and their colocalization in the LH. **(Aa)** Injection site (white dotted lines) of BDA in the SN; **(b)** No BDA-labeled fibers were observed in the DMV; **(c)** The expression of BDA-stained anterograde traced fibers in the LH; **(d)** The magnified areas of the white dotted boxes in **(c)**. **(B–F)** Representative confocal photomicrographs of double-immunofluorescence of TH (green) and D1 (red), TH (green)and D2 (red), OXA (green), and TH (red), D1 and OXA, D2, and OXA in the LH. **(d)** Shows a magnified area of the white dotted box in **(c)**. Scale bars in **(B–F)**: 50 μm. TH, tyrosine hydroxylase; D1, dopamine 1 receptor; D2, dopamine 2 receptor; OXA, orexin A.

The anterograde tracer BDA was microinjected into the left SN to determine whether BDA-labeled fibers could be observed in the DMV. After 7 days, a dense BDA stained area was located at the injection area, the left SN pars compacta (Figure 1Aa). Dense puncta and irregular curved BDA positive nerve fibers were observed in the LH (Figure 1Ac,d). However, no BDA positive fibers were found in the DMV (Figure 1Ab).

Double-label immunofluorescence was performed to assess the distribution patterns of OXA, TH, D1, and D2 in the LH. The results suggested that a considerable quantity OXA-IR, D1-IR, and D2-IR neurons were clearly observed throughout the LH and surrounded by TH-IR fibers. The TH-IR fibers were punctiform or short-bar in shape, and the distribution was not arranged in any particular manner (Figure 1B–D). The OXA was expressed in cytoplasm. We observed that OXA expression was cuneate, gracile, deltoid, buninoid or oval in shape (Figure 1Db,Ea,Fa). In the same slice, D1 expression displayed a similar shape as OXA (Figure 1Bb,Eb), while D2 expression was buninoid (Figure 1Cb,Fb). The numbers of D1-IR, OXA-IR and double-labeled neurons in the LH (*n* = 3) were 1206, 969, and 895, respectively, which were counted from nine sections of three rats in total. The double-labeled neurons accounted for 74.2% of total D1-labeled neurons and 92.3% of total OXA-labeled neurons. However, only a few neurons were both D2-IR and OXA-IR (Figure 1F). The numbers of D2-IR, OXA-IR and their double-labeled neurons in the LH were 1289, 1116, and 334, respectively. The double-labeled neurons accounted for 25.9% of total D2-labeled neurons and 29.9% of total OXA-labeled neurons.

### Decreased Expression of D1 and OXA in the LH of 6-OHDA Rats

**Figure 2:**
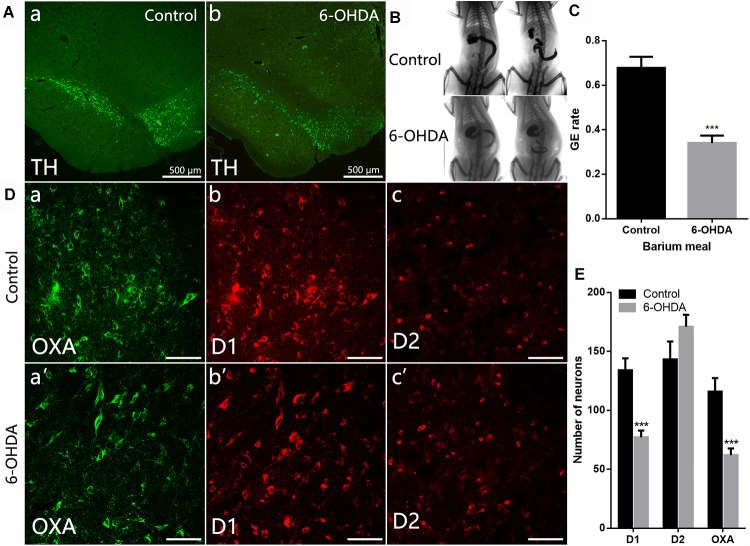
Decreased expression of D1 and OXA in the LH of 6-OHDA rats. **(A)** Expression of TH-IR neurons within the SN in control and 6-OHDA rats; **(B)** Representative images of gastric emptying (GE) at 0 and 90 min after a barium meal in control and 6-OHDA rats; **(C)** GE of barium meals was significantly delayed in 6-OHDA rats compared to control animals; **(D)** Representative alterations in D1, D2, and OXA expression within the LH in control (upper panel) and 6-OHDA rats (lower panel); **(E)** Summary histogram shows a significant decrease number of D1-IR or OXA-IR neurons in the LH, while no significant change of D2-IR neurons in 6-OHDA rats. Scale bars in **(D)**: 50 μm. ^∗∗∗^*P* < 0.001.

Distribution of TH-IR neurons in the SN was detected in control and 6-OHDA rats. TH-IR neurons in the SN was considerably reduced in 6-OHDA rats compared with control ones (Figure 2A). We further evaluated gastric emptying using an *in vivo* digital X-ray imaging system. The results showed that 67.78 ± 5.0% of the stomach contents were emptied at 90 min in control rats. Meanwhile, only 34.01 ± 3.3% was emptied in the 6-OHDA rats (*n* = 6, *P* < 0.001) (Figure 2B,C).

Compared with control rats (Figure 2D,E), the number of D1-IR neurons decreased from 134.0 ± 10.14 to 77.11 ± 5.69 in the 6-OHDA rats (*n* = 3, *P* < 0.001). The number of OXA-IR neurons was also decreased from 111.6 ± 10.38 to 62.00 ± 5.68 (*n* = 6, *P* < 0.001) in the LH of 6-OHDA rats. However, the change of D2-IR neurons number was not significant (*n* = 3, *P* = 0.14), slightly increased from 143.2 ± 15.21 to 170.9 ± 10.14 in 6-OHDA rats.

### Reduced Expression of OX1R and ChAT Protein in the Dorsal Medulla of 6-OHDA Rats

**Figure 3:**
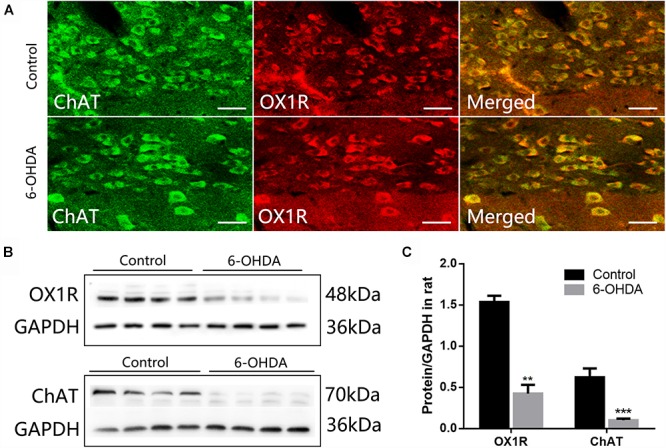
Reduced expression of OX1R and ChAT protein in the dorsal medulla of 6-OHDA rats. **(A)** Colocalization of OX1R and ChAT in the DMV neurons; **(B)** Representative Western blot of OX1R and ChAT in the dorsal medulla from control and 6-OHDA rats. GAPDH was used as a loading control; **(C)** Summary histogram shows that the OX1R and ChAT expression in the dorsal medulla was significantly reduced in 6-OHDA rats. Scale bars: 100 μm. ^∗∗^*P* < 0.01; ^∗∗∗^*P* < 0.001.

ChAT-IR neurons were densely distributed throughout the DMV, and the distribution was not arranged in any particular manner. Nearly all ChAT-IR neurons were also OX1R-IR (Figure 3A). Western blot results showed a significant decrease in the level of OX1R and ChAT protein in the dorsal medulla of 6-OHDA rats, from 1.53 ± 0.07 to 0.42 ± 0.10 (*n* = 4, *P* < 0.001) and 0.62 ± 0.10 to 0.10 ± 0.01 (*n* = 4, *P* < 0.01), respectively (Figure 3B,C).

## Discussion

Most PD patients experience GI dysfunction especially the gastroparesis (Goetze et al., 2006), but its underlying mechanism is not clear. Our previous study has demonstrated that the neurons in the SN could project into the LH and the neurons in the DMV receives the neural projection from the LH (Wang et al., 2014). We speculate that this SN-LH-DMV pathway could modulate the gastric motility since the important role of LH and DMV in the regulation of gastric motility (Travagli et al., 2003, 2006; Stuber and Wiser, 2016). In the present study, we have demonstrated that a considerable quantity of D1 and few D2 was expressed in the OXA-positive neurons in the LH, and OX1R was expressed in the cholinergic neurons of the DMV.

Here we significantly extend our understanding of the SN-LH-DMV pathway by showing that (1) a considerable quantity of D1 and D2 was expressed in the LH, and the OX1R was expressed in the DMV; (2) Nearly all of the D1-IR neurons were also OXA-positive, while only a few neurons expressed both D2 and OXA in the LH, meanwhile the DR-positive neurons were surrounded by the catecholaminergic neural fibers; In the DMV, OX1R was colocalized with ChAT-labeled motor neurons; (3) When dopaminergic neurons in the SN were destroyed, the decreased expression of D1 and OXA in the LH, and the reduced OX1R and ChAT expression in the DMV were observed. These findings suggest that neurons from the SN might regulate the function of OXA-positive neurons in the LH via D1 receptor. The dysfunction of the OXA-positive neurons in turn affects the motor neurons in the DMV through OX1R, ultimately leads to the gastric dysmotility. This study provides a morphologic possibility for the SN-LH-DMV pathway in regulating gastric movement.

LH is one of the functional zones in the hypothalamus, and plays an important role in regulating feeding, sleep and wakefulness (Saper, 2002). A lot of OXA-IR neurons exist in the LH, and most of them are lost in the progression of PD (Thannickal et al., 2008). However, the underlying mechanism regulating OXA release from LH remnant neurons in PD patients is not clear. The SN-LH-DMV neural pathway has been observed in our previous study. In the present study, co-labeling of D1 with OXA in the LH provides an evidence that OXA-IR neurons in the LH might be regulated by the dopaminergic fibers from the SN via D1 receptors, which is further confirmed by surrounding dopaminergic fibers located around the D1-IR neurons in the LH. Moreover, OX1R was highly expressed in the DMV neurons. Specifically, it was presented in retrograde labeled preganglionic neurons from the DMV innervating the stomach. OXA can excite neurons by binding to OX1R of the DMV in rats (Krowicki et al., 2002). The OXA plays a stimulatory effect on the gastric emptying in rats (Ehrstrom et al., 2005; Bulbul et al., 2010b). These data support our idea that lesion of dopaminergic neurons in the SN impairs gastric motility might be mediated by the SN-LH-DMV pathway, in which OXA is a connecting factor between the LH and the DMV.

In the present study, after the dopaminergic neurons in the SN are destroyed by the 6-OHDA, the expressions of both D1 and OXA in the LH, and OX1R and ChAT in the DMV were significantly decrease, suggesting that the excitatory effect from the SN on OXA-positive neurons of the LH and in turn on the vagal cholinergic motor neurons of the DMV would be lowered, which subsequently resulted to gastroparesis in 6-OHDA rats. D1 receptor is a classic subtype of DR family that belongs to G protein-coupled receptors. D1 activates adenylyl cyclase and upregulates intracellular cAMP signaling pathway, whereas D2 inhibits the adenylyl cyclase and downregulates cAMP levels (Baldessarini and Tarazi, 1996). It seems that the decreased D1 in the LH and OX1R in the DMV, respectively, contribute to attenuated OXA and acetylcholine release, respectively. However, detailed mechanism of alteration of the D1, OX1R, OXA, and ChAT needs to be further studied.

In summary, our present study demonstrates that the down-regulated D1 and OX1R might be involved in the process of gastroparesis in PD through the SN-LH-DMV pathway. The SN-LH-DMV pathway has a potential effect on regulating gastric motility.

## Data Availability

All datasets generated for this study are included in the manuscript and/or the supplementary files.

## Author Contributions

J-XZ and Z-YW designed the research and revised the paper. Y-LY performed the experiments and wrote the manuscript. Z-YW, LZ, and YH helped the data analysis. Q-QC, X-RR, and YL provided technical support. J-XZ, L-FZ, and LZ edited the manuscript.

## Conflict of Interest Statement

The authors declare that the research was conducted in the absence of any commercial or financial relationships that could be construed as a potential conflict of interest.
